# Music performance anxiety: evolutionary, developmental, and psychodynamic perspectives

**DOI:** 10.3389/fpsyg.2026.1787319

**Published:** 2026-04-01

**Authors:** Dianna Theadora Kenny

**Affiliations:** The University of Sydney Association of Professors, Darlington, NSW, Australia

**Keywords:** aetiology, children and adolescents, developmental, music performance anxiety, psychodynamic

## Abstract

Music performance anxiety (MPA) occurs in very young children and is prevalent throughout the lifespan of musicians. Childhood presentations are phenotypically similar to adult musicians which raises the question as to whether MPA is innate or acquired and if identified in childhood, the most appropriate way to manage it to forestall MPA as a lifelong problem. An understanding of developmental and psychodynamic psychology and the multifactorial causation of MPA is necessary to develop effective interventions. In this paper, I propose a three-stage model comprising a developmental–relational substrate which, if faulty, results in the development of structural vulnerabilities such as low self-efficacy and maladaptive perfectionism, which in turn triggers performance-activated cognitive–affective processes that underpin behavioral and somatic manifestations of MPA. This model accounts for the appearance of the MPA complex in very young children and its persistence across the lifespan even in elite musicians in the absence of performance catastrophes.

## 1 Introduction

Music performance anxiety (MPA) has been documented in very young children, including 3–4-year-old preschoolers (Boucher and Ryan, 2011), 8–12-year-olds (Ryan, 2005; Tardif et al., 2024; Urruzola and Bernaras, 2020), and adolescents (Fehm and Schmidt, 2006; Osborne and Kenny, 2008; Patston and Osborne, 2016). These and many other studies (e.g., Dias et al., 2025; Kenny and Osborne, 2006; Osborne et al., 2005; Ryan and Andrews, 2021a; Ryan et al., 2021b, 2023; Sims and Ryan, 2024) describe similar symptom complexes in young musicians compared with adult and professional musicians in the following domains:

- Family/social (e.g., attachment quality, early interactions and relationships, parental musical experience, support and expectations, teacher-student relationship, and pedagogical practices) (Kirsner et al., 2023; Ryan et al., 2021b).
- Developmental (e.g., age related maturational changes, performance experience, cognitive and motor skill acquisition, musicality, motivation) (De Lima et al., 2024; Dempsey and Comeau, 2019).
- Individual/psychological (e.g., trait anxiety, self-efficacy, perfectionism, negative affect, psychological vulnerability, attachment insecurity) (Chapman, 2025; Juan, 2025).
- Somatic (e.g., excessive autonomic arousal affecting motor control). Somatic discomfort is a core component for some but not all musicians (Jha et al., 2022; Yoshie et al., 2009).
- Cognitive (e.g., self-focused attention, explicit monitoring, fear of negative evaluation, negative self-talk, catastrophizing). Social/evaluative anxiety triggers maladaptive perfectionism with accompanying explicit monitoring as a coping strategy that robs attentional resources from the performance (Zhang et al., 2023).
- Skill deficits (e.g., technique and task mastery, repertoire choice, practice quality, performance preparation) (Ryan et al., 2023).
- Situational (e.g., type of performance, performance setting, audience, stakes, e.g., competition for scarce resources) (Hatfield and Williamon, 2025; Miles, 2020). Social presence per se elevates arousal which may alter the balance between challenge and threat appraisals (Rey et al., 2025).

In this paper, I explore the theoretical implications of the early appearance and comparable phenotype of the MPA complex in very young and older accomplished musicians.

## 2 Method

This review synthesized empirical literature examining whether MPA reflects innate temperamental vulnerabilities, learning processes acquired through socialization, or a combination, and situational factors that amplify existing vulnerabilities. Electronic searches of PsycINFO, PubMed, Web of Science, and Scopus were conducted for peer-reviewed articles using combinations of the terms *music performance anxiety*, *stage fright*, *temperament*, *behavioral inhibition*, *trait anxiety*, *negative affect, self-efficacy, perfectionism*, *parenting*, *social learning and conditioning*, *early adverse experiences, and child, adolescent and adult musicians*. Reference lists of key papers were also screened. Studies were included if they specifically addressed MPA and examined genetic, neurobiological, temperamental, developmental, or environmental contributors. Findings were synthesized narratively. Evidence was organized under developmental origins (innate or acquired), environmental/situational, and pre-dispositional/characterological frameworks informed by a diathesis-stress model.

## 3 Is MPA innate or acquired?

The commonalities in MPA presentation between child and adult musicians suggest that we need to adopt an evolutionary/developmental/lifespan perspective to aid our understanding of the aetiology and manifestations of MPA. Boucher and Ryan (2011) explored anxiety responses in 63 3–4-year-old children who had undertaken group music lessons and then performed in two concerts. Self-report of anticipatory anxiety using adapted measures suitable for preschoolers, cortisol secretion, and observation of anxious behaviors were assessed. Children with prior performing experience reported less anticipatory anxiety but had higher cortisol levels compared with those without prior experience. Mitigating factors included familiarity with the performance venue and second performances of the same concert. The authors argued that if MPA were innate, in the sense that humans have a biological disposition to feel anxious under conditions of exposure/evaluation, they should experience MPA from the very first performance. If acquired through performance experiences, we would see a difference in the severity of MPA between children who have had previous performance experience and those who have not. Results indicated that the first musical performance was more anxiety provoking than a subsequent occurrence within a short period suggesting that performance anxiety may have both innate and acquired components. Similarly, using the state form of the *State–Trait Anxiety Inventory for Children* (STAIC), 173 children aged 8–12 had significantly higher state anxiety on music performance days compared with non-performance days (Ryan, 2005). The severity of anxiety correlated with children’s baseline trait anxiety scores, indicating that children with higher general anxiety had stronger performance-day anxiety responses. Another study (Urruzola and Bernaras, 2020) of 8–12 year old students concluded that MPA is measurable and varies by age, self-confidence, and fear of negative evaluation even in pre-adolescent children. Tardif et al. (2024) also reported significant physical and emotional manifestations of MPA (stomach butterflies, heart palpitations, and fear of making mistakes before a recital) in 9–12 year old students attending an intensive music program.

The seeds of MPA may be sown even before children commence their formal musical training, especially for children with stable temperamental and personality traits such as a biological predisposition or vulnerability to high trait or generalized anxiety, negative affect, behavioral inhibition, and harm/threat avoidance. These traits are heritable [e.g., there is a shared genetic risk for social anxiety disorder (Scaini et al., 2014) as are hyper-reactivity of threat-detection systems such as amygdala and autonomic nervous system sensitivity (Pereira et al., 2017)]. These traits are observable early in development, although a family history of anxiety disorders exposes the child to familial modelling (i.e., social learning) of anxiety behaviors. Thus, physiological markers commonly observed in MPA—such as heightened sympathetic arousal, cortisol reactivity, and attentional threat bias—are consistent with innate differences in stress responsivity.

To date, there have been no prospective, longitudinal studies of the development of MPA although cross-sectional studies point to the importance of developmental musical experiences from early to late childhood and adolescence. One study (Osborne and Kenny, 2008) reported that MPA in adolescent musicians was best predicted by trait anxiety and gender, but the presence of negative cognitions in their descriptions of their worst musical experience improved the prediction of MPA over trait anxiety and gender alone, highlighting cognitions as an important (acquired) element to address in the treatment of MPA in young musicians. Patston and Osborne (2016) administered measures of MPA—*Music Performance Anxiety Inventory for Adolescents* (MPAI-A; Osborne et al., 2005)—and perfectionism—*Child Multidimensional Perfectionism Scale* (C-MPS; DeKryger, 2005) that assesses concern over mistakes, organization, parental expectations, and doubts about actions—to 526 students (female *n* = 235) aged 10–17. The authors reported strong positive associations between MPA and perfectionism, in particular concern over mistakes, that increased with age and years of experience. The stronger associations for children with greater musical experience perhaps reflects higher discernment or investment. Perfectionism in children aged 7–12 years shares similar dimensions with established factors among adults, and is related to psychopathology even at early ages (DeKryger, 2005).

Like perfectionism, self-efficacy is acquired. Its direction (high/low) is underpinned by the presence of temperamental personality traits and early life experiences. Bersh (2022) reported an inverse relationship between MPA and music self-efficacy in late childhood, i.e., the lower one’s confidence in oneself as a musician, the higher one’s MPA. Consistent with social cognitive theory (Bandura, 2001), mastery experiences and supportive pedagogy predicted lower MPA.

Thus, we may conclude that biological and psychological vulnerabilities create a latent risk for MPA even in young children, which is activated, amplified, or attenuated by learning and situational contingencies. The moderate stability of MPA across the lifespan points to its trait-like quality and cautions us to act early in its mitigation. Longitudinal studies will greatly assist in this endeavor.

### 3.1 Developmental origins of MPA

**Figure 1:**
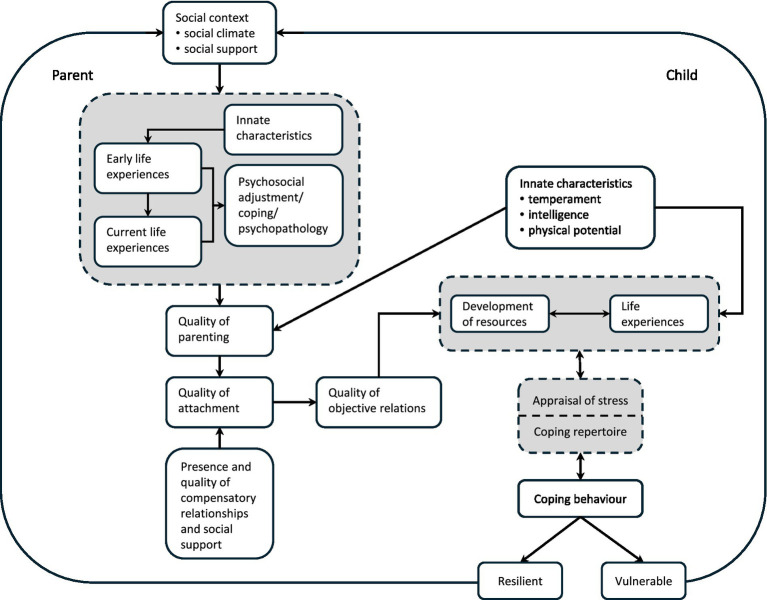
Developmental model of resilience and vulnerability (Reprinted with permission from Kenny, 2000).

Developmental psychology has a great deal to offer our understanding of MPA that occurs to a level of severity beyond the evolutionary mechanisms that had survival value in our primitive past. Experience leads us to adopt different social motivational systems such as caring and cooperative or competitive, combative, and individualistic. We can see how such systems may affect performers’ perceptions of a musical performance, either as a caring interchange between performer and audience or as a competitive, combative interchange where the audience is perceived as a threat that must be withstood. Our habitual “social mentality” directs our perceptions of and behaviors towards our social world. I have explained the development of this social mentality in the context of understanding how children learn to cope with challenges, the outcome of which is either resilience or vulnerability (Kenny, 2000) (see Figure 1).

The developmental theory of coping includes an assessment of the quality of attachment that is determined by the quality of parenting and by the presence and quality of compensatory relationships and/or experiences that were available to the child at critical periods in development. Object relations (i.e., internal working models of relationships) and available resources, both material and personal, determine the way in which life experiences are appraised, and these factors underpin the coping repertoire the child develops. From this repertoire, emotional and behavioral attempts to cope with challenges emerge, and the outcome of this coping behavior is either resilience (positive coping under conditions of risk) or vulnerability (maladaptive coping, including the development of psychopathology). A family or teaching environment characterized by limited opportunity for personal control is associated with the development of anxiety. Building a positive learning history in childhood by providing opportunities to cope adaptively with appropriately calibrated challenges should immunize children against the development of excessive anxiety in response to subsequent challenges. The history of conditioning experiences and their outcomes are a necessary component to consider in the aetiology of anxiety disorders. A third stage, in which specific environmental experiences become conditioned in specific situations is necessary for the development of non-generalized and specific (social) anxiety and, by extension, MPA (Barlow, 2002; Field, 2006).

#### 3.1.1 Situational determinants

Research into MPA in young children opens a broader discussion of the meaning of maladaptive responding under conditions of stress. Two very different models occur in the literature—maladaptation as “disease” or psychopathology and maladaptation as an outcome of aversive developmental experiences, that is, as an interplay of risk and protective factors occurring over time. Patterns of maladaptation may be adaptive responses to maladaptive environments. Managing environments and reducing dynamic risks are important for young performers whose enjoyment and aspirations regarding their art may be derailed early through failure to address key issues such as the role of parents and teachers in precipitating and maintaining anxious responding, and pedagogical methods and institutional teaching contexts such as pace of instruction, selection of appropriate repertoire, establishment of effective practice routines, provision of minimally stressful early performance experiences, reduction of the emphasis on competitions, and encouragement a balanced lifestyle (Barros et al., 2022; Jeong and Ryan, 2022).

Clinical history taking of anxious musicians highlights a common cascade of early negative performance experiences like exposure to critical or punitive parenting/teaching style resulting in maladaptive cognitive schemas (i.e., catastrophizing and fear of negative evaluation), performance errors followed by shaming and humiliation, loss of self-efficacy, and the internalization of maladaptive perfectionistic standards. Through these conditioning experiences, performance contexts become conditioned stimuli for MPA, in conjunction with innate temperament and neurobiology, with more vulnerable individuals more likely to develop MPA under conditions of lower stress.

From the foregone discussion, MPA may be conceptualized within a diathesis-stress framework in which innate vulnerable predispositions interact with situational variables in mutually amplifying feedback loops that in an extreme form leads to repeated performance breakdown and eventually, for some, leaving the field.

In addition, we must not ignore context-dependent variability such as music genre. For example, classically trained musicians tend to be more self-oriented during performance and report fewer positive performance experiences compared with non-classical musicians (Perdomo-Guevara, 2014). Consider the following case example of a young musician I consulted for severe MPA.

Amelie, a 16-year-old student in senior high school had chosen an advanced music course as part of her study program for her high school diploma. There were two streams – classical and contemporary. Amelie’s singing teacher placed her in the classical stream because she had studied classical singing from a young age and had a “beautiful voice.” Amelie subsequently developed increasing MPA that significantly impaired her performances. She reported loss of volume, voice projection, and breath control, and tension in her oral motor musculature. She also complained about insecurity in her voice when changing registers and difficulty in hitting the higher notes in her repertoire. She had received 10 sessions of cognitive behavior therapy (CBT) and several sessions with a physiotherapist to reduce vocal tension with no effect. She was then referred to me. It was immediately apparent that her parents were far too invested in Amelie’s performances. They attended every recital and would not speak to her for several days after a sub-standard performance. Amelie told me that she had wanted to join the contemporary singing stream but had not been asked for her preference. We discussed the concept of voice change, and I explained that teenage girls aged 10 and 16 undergo adolescent voice change during which the larynx enlarges, and the vocal cords thicken. Singing through voice change can make the voice feel unstable. This was one of the main causes of Amelie’s MPA compounded by her parents’ over involvement and high expectations and her singing teacher not allowing her to choose her preferred stream. Discussions with the parents to reduce their attendance and involvement in Amelie’s singing and discussions with the singing teacher to allow her to change to the contemporary stream led to a rapid resolution of Amelie’s MPA.

### 3.2 Self-efficacy and perfectionism

Just as for adult musicians, there are strong associations between MPA in adolescent musicians and personality traits such as optimism, stress propensity (trait anxiety), achievement motivation (Stoeber et al., 2007), sensitivity to reward and punishment (Alzugaray et al., 2016; Dempsey and Comeau, 2019; Taft, 2019), self-esteem, self-efficacy, and perfectionism (Dobos et al., 2019) that may be understood as outcomes of developmental experiences (Kenny, 2011; Kenny and Osborne, 2006). Many papers on MPA in young people include measures of self-efficacy and perfectionism, mostly without a dynamic description of their development. Few recognize the overlap in aetiology and manifestation outside of reporting strong correlations between them and their inverse relationships with MPA (Egilmez, 2015).

Perfectionism of the maladaptive kind involves setting unrealistically high standards and being overly critical of mistakes, a construct closely aligned with fear of negative evaluation, with temporality the main distinguishing feature (the latter anticipatory, the former *post hoc*) (Gök and Yalçınkaya-Alkar, 2025).

Musically, maladaptive perfectionism leads to over-practising, intense fear of the performance context, and severe MPA. This contrasts with “high achievers” whose standards are flexible and attainable, who can learn from mistakes without collapsing in shame, and whose self-worth is stable in the face of setbacks. In maladaptive perfectionism, standards are rigid and absolute, failure threatens the sense of self, pride is fleeting, and self-worth is conditional upon reaching the imagined required standard. Exaggerated perfectionism is linked to feelings of being a sham or impostor. As one musician told me:

For me as a professional musician, a wrong note is a catastrophe, a wrong note is an indicator that I’m not as good as I say I am.

**Figure 2:**
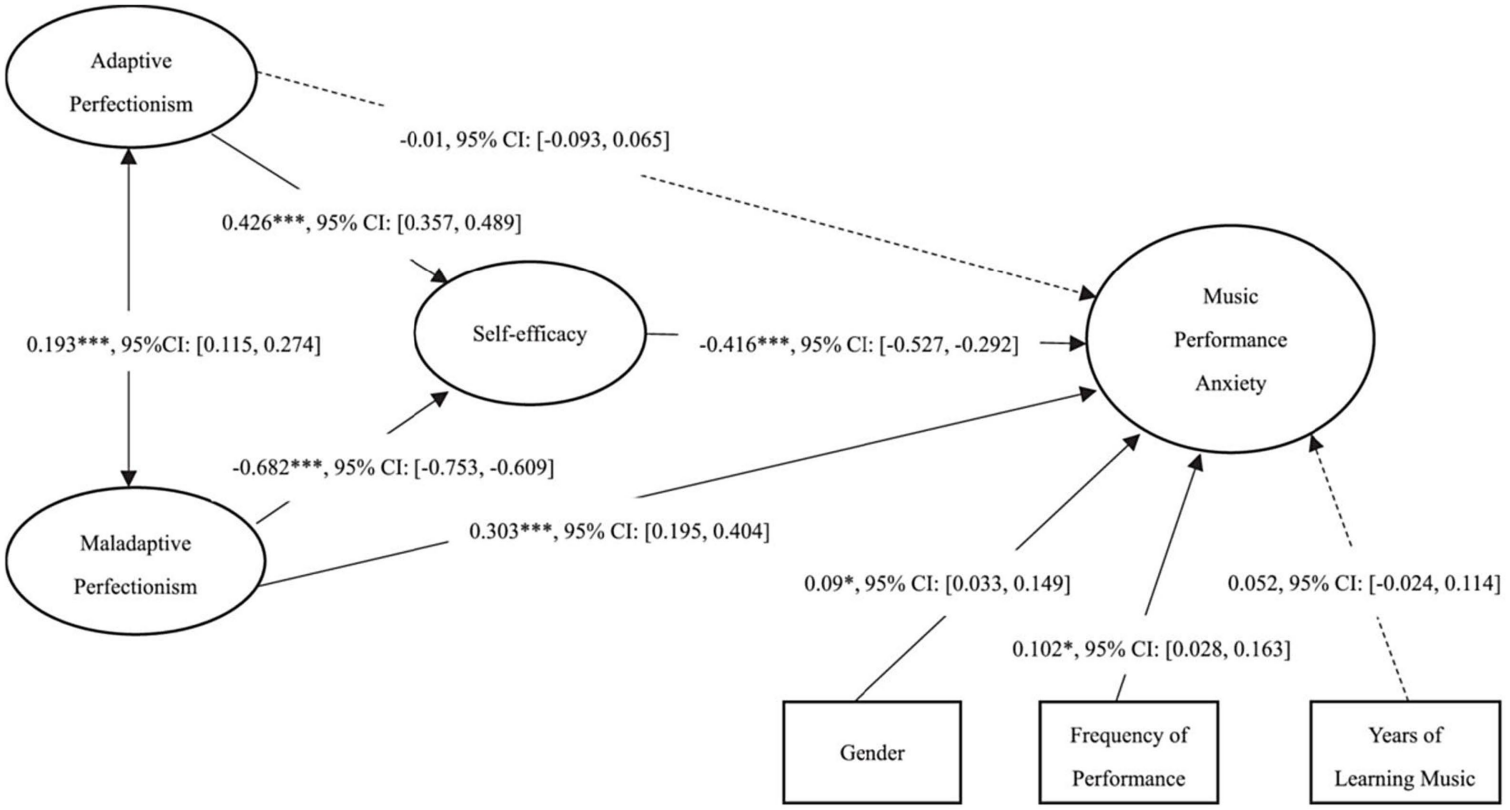
Indirect effects model with bias-corrected bootstrap test on mediating effects. [Dashed line indicates the predictive path was nonsignificant. *p < 0.05, ***p < 0.001] (Reprinted with permission from He and Ding, 2025).

Both adaptive and maladaptive perfectionism predict MPA through self-efficacy (He and Ding, 2025). Figure 2 demonstrates the correlational relationships between these constructs. It is noteworthy that sex (stated as gender in the figure) and performance frequency only weakly predicted MPA, and years of learning music did not predict MPA at all.

Perfectionistic young people who exhibit unrealistically high demands and expectations of themselves, severe self-criticism, and over concern or intolerance for mistakes are likely to have parents and/or teachers who have been overly invested in the child’s performances and achievements and high, often unrealistic expectations of outcome but with insufficient support or empathy for the child’s “failures.” This creates a generalized psychological vulnerability in the child who internalizes the unacceptability of any sub optimal performance, leading to the development of maladaptive perfectionism that includes low self-esteem, low self-efficacy, and loss of spontaneity and enjoyment in performance due to excessive self-focused attention, excess muscle tension, loss of automaticity, and fear of negative evaluation. This imbues the performance environment with danger and pervasive negativity.

Hence, self-efficacy and perfectionism are intricately dynamically connected in their aetiology and manifestations (Arbinaga, 2023). At the core of each construct is shame or the fear of being shamed by one’s inadequacy. Both low self-efficacy and maladaptive perfectionism reactivate early object relations that trigger appraisals about the nature of the audience as supportive or hostile, the expected judgement as kind or critical, and the experience of fraudulence and shame that evokes catastrophic anticipation of mistakes, somatic anxiety and motor disruption, and excessive self-monitoring that disrupts automaticity (Anton et al., 2019).

Given the commonalities between self-efficacy and perfectionism in aetiology, high inter-correlation, and inverse relationships with MPA, the question as to whether there is a clear empirical boundary between these constructs arises. Available evidence suggests that they are distinct, if overlapping constructs because they demonstrate different patterns of correlation with outcomes, respond differently to interventions, and show distinct factor structures in psychometric analyses. These constructs can co-occur but also appear independently - someone can have high self-efficacy without being perfectionistic or be perfectionistic while having low self-efficacy in specific domains (Flett et al., 2011; Hart et al., 1998). Consider this comment by American singer-songwriter, Walker Hayes:

My dad was listening to me noodle around on the guitar in the house and sing, and he was like, ‘Man, you are funny, and you sound good when you do that. You should do that at a bar.’ I had stage fright, so I was like, ‘No, Dad. Leave me alone.’

As a young performer, Hayes showed good self-awareness—that he was not ready for public performances—and embarking on such before he felt psychologically (and musically) ready would have ruined his enjoyment of his art and perhaps forestalled his musical career. When he did start performing, he said:

For me, a good show is not a perfect show; it’s just one where you connected.

Notwithstanding his wisdom and self-awareness, Hayes started drinking alcohol at the age of 13 to manage his anxiety and descended into alcoholism, becoming sober 23 years later at age 36. Hays did not appear to be a perfectionist, at least cognitively; however, he suffered very severe MPA that he assuaged with alcohol. He described a pervasive “insecurity” about performing and that he used alcohol as his “crutch” to dampen his profound feelings of vulnerability, which strongly suggests low self-efficacy. He grew up in a blended family with eight half-siblings, feeling he “didn’t get enough” from his father, that his father was “hurtful.” He described his insecure, ambivalent attachment: “…grief is like liquid, it is never still, it changes shape and form every day…”

#### 3.2.1 Social psychological and psychodynamic understandings of self-efficacy and perfectionism

It is instructive to consider two dominant theoretical frameworks for understanding the relationships between self-efficacy and perfectionism. In Bandura’s social-psychological theory, self-efficacy refers to an individual’s belief in his/her capacity to organize and execute actions required to achieve desired outcomes. In psychodynamic theory, this belief is not treated as a primarily cognitive appraisal, but as a developmentally constructed sense of agency, rooted in early relational experience and intrapsychic structure. People with high self-efficacy have confidence in their capacity, the ability to reality test, plan, take initiative, persist, effectively control impulses, and tolerate frustration, uncertainty, and failure without collapse. These functions are developed via the internalization (through modelling and imitation) of supportive, reliable objects (i.e., primary caregivers). Both self-efficacy and perfectionism are based on the quality of internalized relationships and are shaped by superego^1^ functions, which can be either benign and supportive or harsh and punitive. Thus, self-efficacy is a structural and affective achievement, not merely an expectation of success (de Serra Queiroz and Andersen, 2020). Attempting a task is never purely instrumental, it is unconsciously relational. Action may evoke fantasies of approval, rivalry, retaliation, or abandonment. Avoidance may protect against internalized object responses rather than external failure. Thus, diminished self-efficacy often masks fear of relational consequences, not incapacity (Bandura, 2001). Low self-efficacy reflects an internal world in which initiative and performance (i.e., exposure to scrutiny) are linked to danger—loss of love or approval, criticism, and shaming (Passanisi et al., 2015).

Low self-efficacy sometimes realistically reflects a lack of skill, at others a fear of self- or other- attack following failure to meet perceived required standards. Those with low self-efficacy engage in defensive behaviors like avoidance to forestall anticipated failure, criticism, and humiliation, and procrastination to manage ambivalence about performing and their uncertainty of success (Klassen et al., 2008).

From a psychodynamic perspective, maladaptive perfectionism is understood as a defensive personality organization rather than a healthy striving for an attainable high standard (adaptive perfectionism) (Cohen, 2020). The central dynamic conflict in maladaptive perfectionism is shame avoidance, expressed as harsh self-criticism following perceived failure to attain unrealistic goals in a pre-emptive strike to forestall the same criticism from others. When goals are not attained, maladaptive perfectionists are left feeling inherently flawed rather than forgivably human. Perfectionistic strivings constitute attempts to compensate for the perceived defects in the self (McWilliams, 2011). This striving for the unattainable represents an attempt to regulate unconscious anxiety, shame, and fear of loss of love and respect through excessive control and achievement. At its core is a fragile and conditional self-concept that has been internalized from unsatisfactory early relational experiences in which parental love was contingent on a specific standard of performance. Perfectionism potentially mediates the relationship between attachment patterns and MPA, suggesting that perfectionism operates through distinct psychological pathways (Rice and Mirzadeh, 2000).

Any young person presenting for therapy with low self-efficacy/maladaptive perfectionism requires an intervention that focuses on the precise psychopathology that must include parents and teachers to modify parenting and pedagogy where required, and an assessment of musical goals to ascertain their appropriateness for the level of aptitude, skill, technical mastery, and motivation in the young musician. Stage two of therapy, if the young person wishes to continue their musical education, firstly exposes the developmental-relational substrate (early attachments, conditional approval in caregiving and pedagogy, and temperamental sensitivity); secondly, with structural factors (self-efficacy and perfectionism etc.); and thirdly, performance-activated cognitive-affective processes (negative self-schemas, self-focused attention, hypervigilance, and catastrophizing). The therapy must work through feelings of shame and fear of exposure as an imposter, feeling like someone not deserving to be on stage playing with truly talented musicians, to support the emergence of a more compassionate, internalized sense of self from whom a perfect performance ceases to be the only source of self-worth or self-esteem. Not every anxious musician will need therapeutic work at all three levels. As a psychotherapist, I tend to work with the most affected musicians and describe each substrate here to explain the different levels of seriousness and impairment that can occur with MPA. Many will respond favorably to systematic work at the second and third levels. Therapeutic work restores self-efficacy and reduces maladaptive perfectionism by modifying the relational meaning of action, not by skills training alone. Put differently, self-efficacy improves not when the person is convinced that they can succeed, but when they believe that trying will not destroy the self or the relationship. Musical self-efficacy depends on whether visibility/exposure is experienced as survivable.

## 4 Discussion

In this paper, I have attempted to account for the presence of MPA in very young children and accomplished musicians by integrating our understanding of the developmental and social-psychological processes that underpin healthy development with our understanding of its manifestation in musicians across the lifespan. This model conceptualizes MPA as a disorder of threatened agency under evaluative exposure, in which self-efficacy collapses due to the activation of internalized relational and shame-based processes. These processes are activated at birth, and account, at least in part, for the manifestation of MPA in children as young as 3–4 years. I term this the developmental-relational substrate of MPA. When the experiences in this developmental domain are sub-optimal, the child develops structural vulnerabilities such as low self-efficacy and maladaptive perfectionism that may lead to performance activated cognitive affective processes such fear of negative evaluation, self-focused attention, explicit monitoring, and catastrophic interpretation of minor errors, procrastination and defensive avoidance. These may be accompanied by behavioral and somatic manifestations of MPA.

In summary, MPA arises when performance activates internalized threatening evaluative relationships that transform musical action into a relational threat. This activation precipitates a collapse of self-efficacy and a triggering of maladaptive perfectionism, expressed phenomenologically as anxiety, cognitively as self-focused attention and explicit monitoring, somatically as performance disruption, and behaviorally as defensive control. This formulation accounts for the emergence of MPA in childhood and adolescence, when young people are undergoing identity formation and social integration. It also accounts for the appearance of MPA in early adulthood after years of training and successful performances, the persistence of MPA throughout the lifespan, and the occurrence of severe MPA even in highly skilled musicians who rarely if ever experience performance breakdowns. It also illuminates the reasons for partial or non-response to cognitive behavior therapy and positive psychology interventions when shame-based superego dynamics remain unaddressed.

## Footnotes

1 The superego is a structural component of the psyche that represents the internalized moral, evaluative, and prohibitive functions of early authority figures (parents, teachers, significant others). It develops through identification and internalization of values and beliefs and then functions to regulate behavior, affect, and self-evaluation in accordance with internal standards rather than external enforcement (Milrod, 2002).
